# Studies on the Physiological Response of *Hemerocallis middendorffii* to Two Types of Drought Stresses

**DOI:** 10.3390/ijms252413733

**Published:** 2024-12-23

**Authors:** Qi Wang, Xi Lu, Yue Sun, Jiahui Yu, Qingtao Cao, Yiting Xiao, Nan Jiang, Lifei Chen, Yunwei Zhou

**Affiliations:** College of Horticulture, Jilin Agricultural University, 2888 Xincheng Street, Changchun 130118, China; w3977362@163.com (Q.W.); luxi@jlau.edu.cn (X.L.); sy1016@jlau.edu.cn (Y.S.); 13234407788@163.com (J.Y.); 15961328355@163.com (Q.C.); xiaoyitingo_0@163.com (Y.X.); jiangnan09160420@163.com (N.J.)

**Keywords:** *Hemerocallis middendorffii*, drought stress, morphological, physiological characteristics, *HmWRKY9*

## Abstract

Drought is a major environmental factor limiting plant growth and development. *Hemerocallis middendorffii* is a perennial herbaceous plant with high drought resistance, and high ornamental and application values. Understanding the mechanism of drought stress resistance in *H. middendorffii* is helpful for better utilization of plant resources and selection of excellent germplasms. In this study, the phenological and physiological traits of *H. middendorffii* were comprehensively analyzed under natural drought stress (ND) and PEG-simulated drought stress (PD), and the resistance of *H. middendorffii* to different levels of drought stress was evaluated. ND was treated using a natural water loss method. PD was treated under drought stress by using PEG-6000. *H. middendorffii* were able to grow within 15 d of ND and 4 d of 20% PD. Beyond this drought time, *H. middendorffii* will wilt and lose their ornamental value. Further study showed that *H. middendorffii* protect themselves from damage and enhance drought resistance mainly by increasing the content of osmoregulatory substances, enhancing the activity of antioxidant enzymes, and inhibiting photosynthesis. Malondialdehyde (MDA) content accumulated rapidly at 15 d of ND and 7 d of PD. Antioxidant enzyme activities peaked at 15 d of ND and 4 d of PD. Photosynthetic parameters decreased at 15 d of ND and 4 d of 20% PD, respectively. Moreover, we identified that the *HmWRKY9* gene was up-regulated for expression in the leaves after ND and PD. *HmWRKY9* may be involved in regulating the response of *H. middendorffii* to drought stress.

## 1. Introduction

Drought is one of the most serious and widespread natural disasters. It affects soil moisture and atmospheric dryness in terrestrial ecosystems, as well as plant growth and development, and may even lead to plant death [1,2]. When plants are subjected to drought stress, complex regulatory mechanisms are activated to alleviate the stress [3]. The phenotype of plants subjected to drought stress is characterized by changes in biomass metrics such as height, crown spread, and leaf length. Plants subjected to drought stress have an efficient rooting system [4,5]. The differential response of roots and stems to drought adversity is generally considered to be a plant adaptation to drought conditions [6]. Sun et al. demonstrated that root elongation facilitates water uptake by plants to resist drought [7]. Drought stress affects the external morphology of plants. In *Saccharum officinarum*, drought stress increased the average internode length and disrupted the intact root structure [8]. In *Matthiola incana*, plant height, stem fresh weight, stem dry weight, root fresh weight, and root dry weight significantly decreased after drought stress [9]. *Cunninghamia lanceolate* increases the complexity and elongation of the root system and reduces the branching angle of the root system. This leads to a steeper and deeper root system that can adapt to drought stress [10].

Photosynthesis is the primary way for plants to obtain energy. Drought also disrupts the photosynthetic system of plants [11]. Under drought conditions, the stomata of leaves are closed and CO_2_ uptake is reduced. The activity of calvin cycle enzymes is decreased, and the ratio of photorespiration to dark respiration is increased. The photosynthetic apparatus is damaged, leading to a significant decline in efficiency [12]. It is mainly reflected by related indexes such as net photosynthetic rate (Pn), stomatal conductance (Gs), intercellular CO_2_ concentration (Ci), and transpiration rate (Tr). Silva et al. showed that drought caused a decrease in plant photosynthesis [13]. Gao et al. studied *Glycyrrhiza glabra* under different drought conditions and found that Tr, Pn, and Gs decreased significantly with increasing drought stress; the photosynthetic capacity of the leaves was reduced; and ΦPSII was partially inactivated [14]. Ma et al. investigated the effect of drought on apple photosynthesis, in which both Pn and Gs showed a decreasing trend [15]. The relative water content (RWC) of leaves provides an indication of the water retention capacity in different plants [16]. Drought stress causes cell membrane lipid peroxidation to produce MDA. MDA reflects the degree of adversity injury and its oxidative stress status in plants and is an important indicator of the degree of adversity injury in plant [17]. It has been shown that drought leads to a decrease in the water content available for light energy utilization by plants and a decrease in water use efficiency. Eventually, photosynthesis in plants will be reduced [18].

Accumulating osmoregulation substances and improving antioxidant capacity are two essential mechanisms for plants to survive drought stress [19]. Under normal conditions, the production and removal of reactive oxygen species in plants is in dynamic equilibrium. This equilibrium is disrupted when plants are subjected to drought stress, which leads to the accumulation of reactive oxygen species and oxidative stress [20]. Li et al. investigated the effects of drought stress on two *Morus multicaulis* species and showed that soluble sugar (SS), proline concentration (Pro) and soluble protein, MDA and SOD levels, and peroxidase (POD) activity were decreased [21]. It has been shown that drought stress significantly affects osmoregulatory substances and antioxidant systems [22]. Plants subjected to drought stress can induce oxidative damage to membranes by increasing the lipid peroxidation level and increasing the activity of oxidizing enzymes to counteract the damage caused by drought in plants [23]. Liu et al. investigated the physiological effects of highland barley under drought stress, and their results showed that artemisinin and drought contributed to the increase in SOD activity and the decrease in POD activity [24].

WRKYs are a unique family of transcription factors (TFs) in plants. WRKYs regulate many abiotic stress responses in plants and are particularly common in drought stress [25,26]. AtWRKY33 was involved in drought stress by directly regulating the expression of *CesA8* [26]. *AtWRKY40* could maintain cellular water uptake by enhancing the osmoregulation and antioxidant capacity of plants [27], thereby increasing their resistance to drought stress [28]. Wang et al. showed that the overexpression of *ZmWRKY40* increased POD and catalase (CAT) activities in transgenic *Arabidopsis* to reduce ROS levels [29]. We obtained transcriptome data of *H. middendorffii* in our previous study and preliminarily identified 41 *HmWRKYs* genes involved in the drought stress response of *H. middendorffii*. After natural drought treatment and PEG-simulated drought treatment, the expressions of *HmWRKYs* in the leaves and roots were analyzed. One up-regulated and nine down-regulated *HmWRKYs* were found in the leaves, and three up-regulated and one down-regulated *HmWRKYs* were found in the roots. The expression of *HmWRKY9* was significantly increased in the leaves of *H. middendorffii* after drought stress, and *HmWRKY9* was identified as a candidate gene for drought resistance.

*Hemerocallis middendorffii* is an ornamental plant with strong drought tolerance. *H. middendorffii* (Asphodelaceae, *Hemerocallis*) is one of the original parents of the modern daylily. It is highly drought-resistant, has colorful flowers, and has second-flowering characteristics. *H. middendorffii* are mainly distributed in East Asia and widely used in landscaping. However, there are fewer studies on the resistance mechanisms of *H. middendorffii* under drought stress. This study aimed to investigate the effects of ND and PD on the physiological characteristics of *H. middendorffii* and to assess the growth of *H. middendorffii* under drought conditions. Moreover, the *HmWRKY9* gene was screened and cloned from the drought stress-related transcriptome of *H. middendorffii*, and its expression under drought stress was determined. This is important for understanding the drought tolerance mechanism and for breeding drought-tolerant *H. middendorffii* species.

## 2. Results

### 2.1. Determination of Leaf Water Content and Morphology of H. middendorffii Under ND

**Figure 1:**
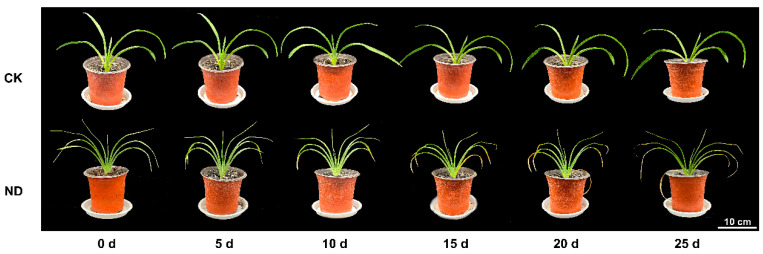
Morphological changes in *H. middendorffii* under different natural drought stresses. The graph was generated based on the morphological response of *H. middendorffii* to stress time (x-axis) after natural drought stress (ND)/control check (CK) (y-axis).

Morphological differences between the plants in the control check (CK) and ND became more and more obvious with the increase in drought duration (Figure 1). Plants in the middle of a drought (5–10 d) were still thriving with little change in morphology and leaf color. In the later stages of drought (15–25 d), ND-treated plants lost luster and appeared yellow and wilted. Increased spacing of leaves resulted in loss of ornamental value of the ND-treated plants. Drought tolerance was graded according to their phenotypes after drought treatment, with 0–5 d being class I, 5–10 d being class II, and 15–25 d being class III (Table 1).

**Figure 2:**
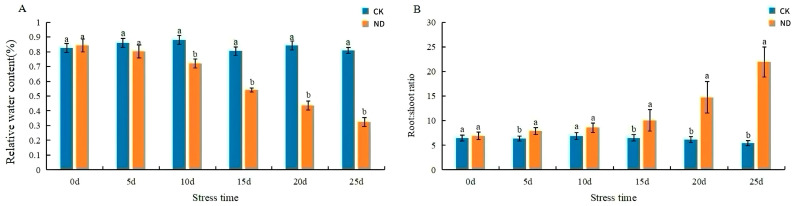
Effects of relative water content and root–shoot ratio of *H. middendorffii* under ND. (**A**) Relative water content was significantly decreased in plants in ND. (**B**) Root–shoot ratio was significantly increased in plants in ND. CK and ND are represented by blue and orange bars, respectively. The stress time (x-axis) represents the degree of ND to which *H. middendorffii* was subjected. Means with different lowercase letters in the same stress time indicate there are significant differences between different treatment groups (*p* < 0.05).

The leaf width of the plants in ND reached a maximum of 15 d (class III) and slowly decreased. Leaf length and width were significantly reduced in ND plants compared to CK, whereas crown width increased by 40.02% (Table 2). The above-ground fresh weight of plants in ND decreased by 45.10% at 10 d (Table 3). The relative water content of CK leaves remained at about 80%, while the relative water content of plants in ND leaves declined rapidly (Figure 2A). The root–shoot ratio of plants in ND increased rapidly (Figure 2B).

### 2.2. Determination of Photosynthetic and Chlorophyll Fluorescence Parameters of H. middendorffii Under ND

**Figure 3:**
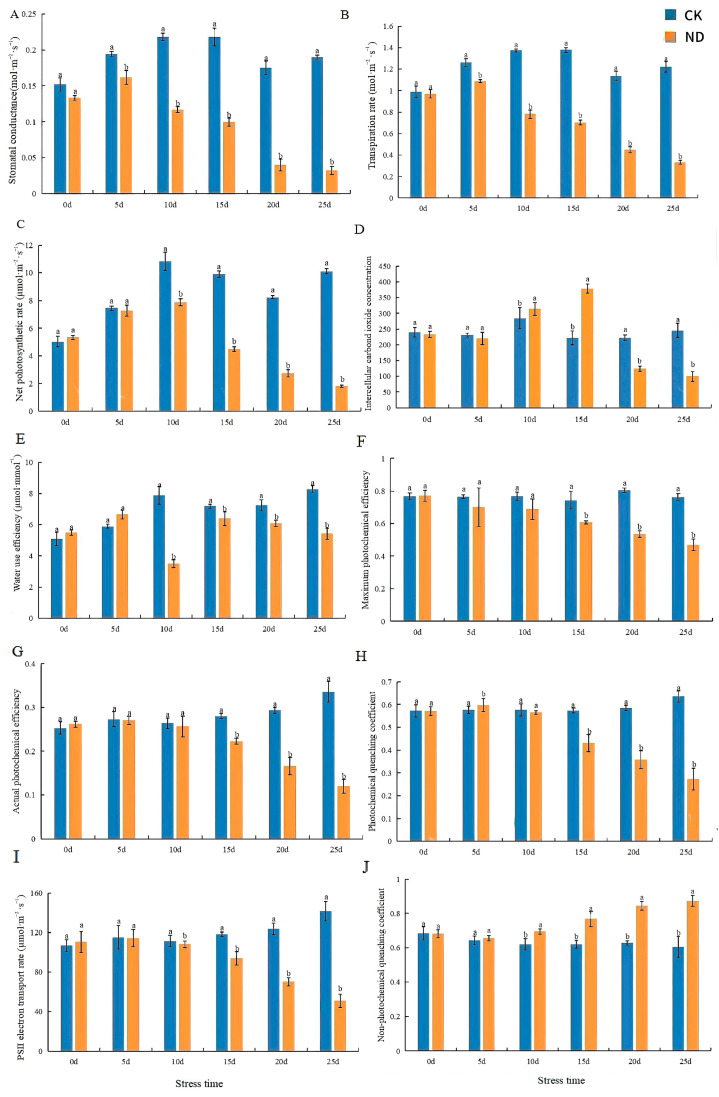
Effects of photosynthetic parameters of *H. middendorffii* under drought stress. (**A**–**J**) Stomatal conductance (Gs), transpiration rate (Tr), net photosynthetic rate (Pn), intercellular CO_2_ concentration (Ci), water use efficiency (WUE), maximum photochemical efficiency of PSII (Fv/Fm), actual photochemical efficiency (ΦPSII), photochemical quenching coefficient (qP), PSII electron transport rate (ETR), and non-photochemical quenching coefficient. The photosynthetic parameters of *H. middendorffii* initially increased and then decreased within 25 d of ND. CK and ND are represented by blue and orange bars, respectively. Means with different lowercase letters in the same stress time indicate there are significant differences between different treatment groups (*p* < 0.05).

Within 25 d of drought stress, compared with CK, Gs and Tr significantly decreased by 72.71% and 82.88%, respectively (Figure 3A,B). Pn reached its highest value at 10 d and its lowest at 25 d (Figure 3C). The decline was most pronounced at 15 d, with a 42.88% decrease from 10 d. Ci reached its lowest value at 232.5163 µmol·mol^−1^ (Figure 3D). Within 25 d of drought stress, exhibited a 53.83% increase in Ci compared to CK.

The Fv/Fm, ΦPSII, qP, and ETR values in *H. middendorffii* seedlings exhibited a decline with an increase in the degree of drought (Figure 3F–I). After 25 d, the most pronounced decline in the Fv/Fm, ΦPSII, qP, and ETR values was observed. They were 38.48%, 64.25%, 57.08%, and 64.26% lower than the CK Fv/Fm, ΦPSII, qP, and ETR, respectively. The qN increased by 44.03% compared to the CK after 25 d (Figure 3J).

### 2.3. Determination of Cell Membrane Systems, Chlorophyll Concentration, Osmotic Adjustment Substances, and Antioxidant Enzyme Activity of H. middendorffii Under ND

**Figure 4:**
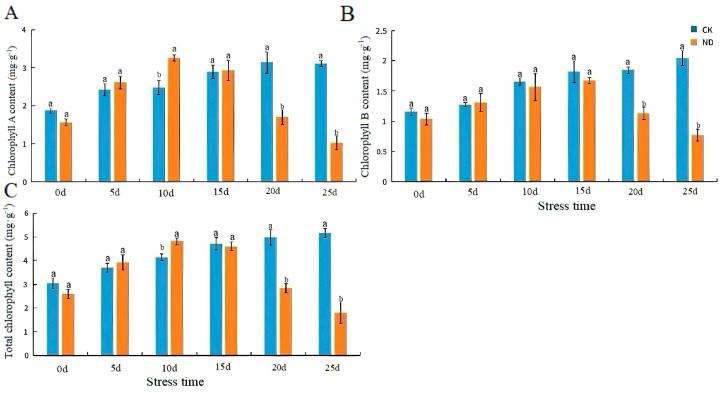
Effects of chlorophyll fluorescence parameters of *H. middendorffii* under drought stress. (**A**–**C**) Chlorophyll A, chlorophyll B, and total chlorophyll. The chlorophyll content of *H. middendorffii* initially increased and then decreased within 25 d of ND. CK and ND are represented by blue and orange bars, respectively. Means with different lowercase letters in the same stress time indicate there are significant differences between different treatment groups (*p* < 0.05).

The chlorophyll and total chlorophyll contents of plants in ND reached their maximums at 10 d (Figure 4A), at 3.25 mg·g^−1^ and 4.82 mg·g^−1^ (Figure 4C), respectively.

**Figure 5:**
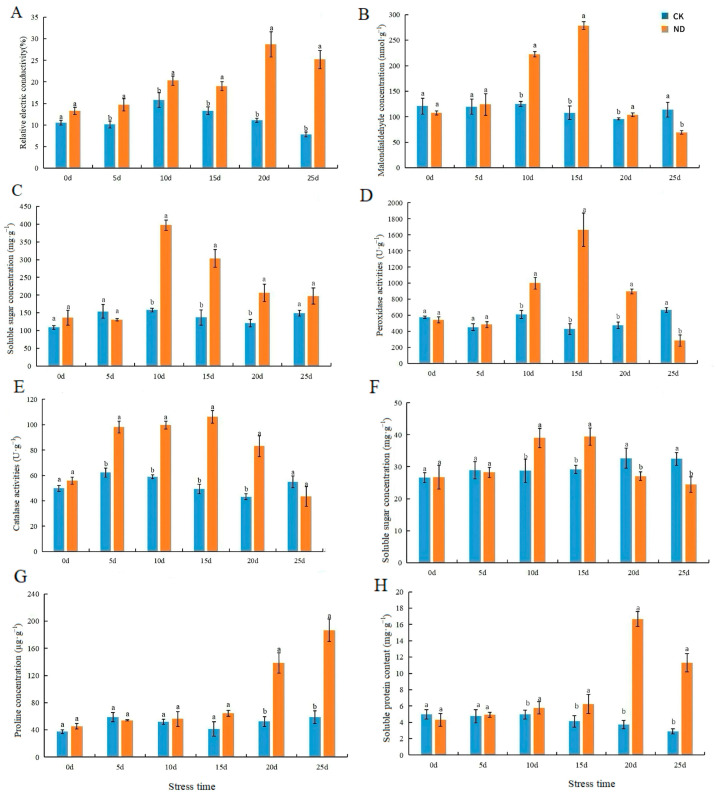
Effects of antioxidant enzyme activities and concentration of osmotic adjustment substances of *H. middendorffii* under ND. (**A**–**H**) Relative electric conductivity, malondialdehyde (MDA) concentration, superoxide dismutase (SOD) activities, peroxidase (POD) activities, catalase (CAT) activities, soluble sugar (SS) concentration, proline concentration (Pro), and soluble protein content. Osmoregulatory substances and antioxidant enzyme activities in *H. middendorffii* leaves were consistently elevated within 15d of ND. CK and ND are represented by blue and orange bars, respectively. Means with different lowercase letters in the same stress time indicate there are significant differences between different treatment groups (*p* < 0.05).

The MDA content of plants in ND peaked at 278.49 nmol·g^−1^ at 15 d, an increase of 158.74% compared to CK (Figure 5B). Compared with CK, the SOD activity suddenly increased from 10 to 15 d of the plants being in ND by 260 U·g^−1^ and 145.55 U·g^−1^, respectively (Figure 5C). Moreover, SOD activity reached its peak in 10 d. The POD activity of CK peaked at 1664.37 U·g^−1^ in 15 d (Figure 5D). CAT activity reached a maximum value of 106.13 U·g^−1^ in 15 d and a minimum of 43.46 U·g^−1^ in 25 d (Figure 5E). The Pro content of plants in ND tended to increase in general (Figure 5G).

### 2.4. Determination of Leaf Water Content and Morphology of H. middendorffii Under PD

**Figure 6:**
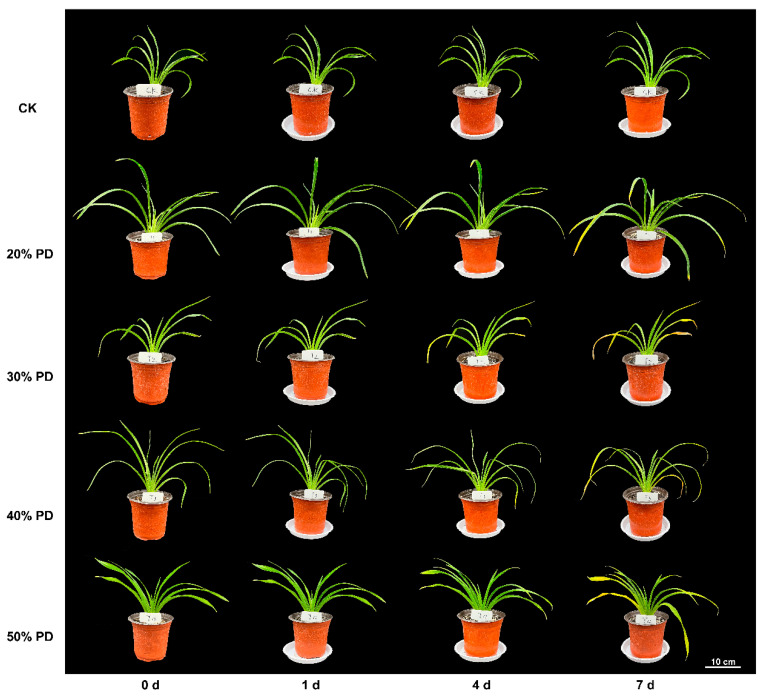
Morphological changes in *H. middendorffii* under different PEG stresses. The graph was generated based on the morphological response of *H. middendorffii* to stress time (x-axis) under different concentrations of PEG-6000 drought stress (PD)/control check (CK) (y-axis).

PEG simulation of drought stress (PD) is a standard experimental method (Figure 6). Plants in 20% PD showed little morphological change during 7 d of stress. At the late stage of drought (4–7 d), the leaves of plants in 30% PD, 40% PD, and 50% PD showed apparent yellowing and wilting, with increasing leaf spacing, and the plants lost their ornamental value. Drought tolerance was graded according to their phenotypes after PD: 20% PD, 30% PD, 40% PD, and 50% PD (0–1 d) were class I; 20% PD (1–7 d) were class II; 30% PD, 40% PD, and 50% PD (1–4 d) were class II; and 30% PD, 40% PD, and 50% PD (4–7 d) were class III (Table 1).

Within 7 d of PEG stress, the plant height of PD and CK gradually increased. After 4 d of PD, there was a significant difference in leaf width between 50% PD and CK. The crown amplitude of 20–40% PD increased significantly. As the concentration of PEG increased, leaf dehydration and wilting became more severe, resulting in smaller leaf widths (Table 4).

### 2.5. Determination of Photosynthetic and Chlorophyll Fluorescence Parameters of H. middendorffii Under PD

**Figure 7:**
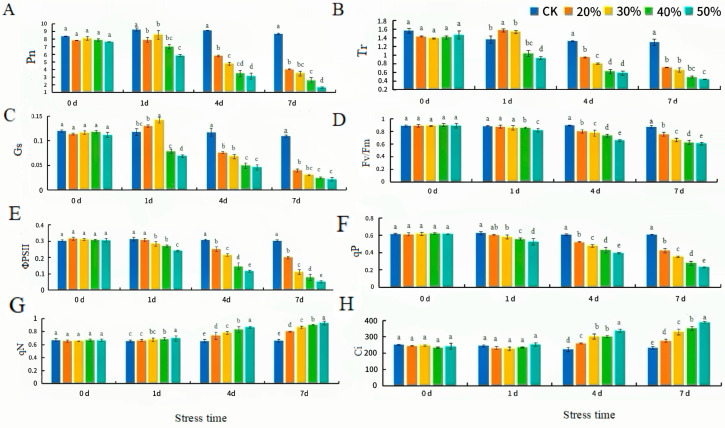
Photosynthetic parameter changes in *H. middendorffii* under PD. (**A**–**H**) Net photosynthetic rate (Pn), transpiration rate (Tr), stomatal conductance (Gs), maximum photochemical efficiency of PSII (Fv/Fm), actual photochemical efficiency (ΦPSII), photochemical quenching coefficient (qP), non-photochemical quenching coefficient (qN), and intercellular CO_2_ concentration (Ci). The photosynthetic parameters of *H. middendorffii* initially increased and then decreased within 7 d of 20% PD and 30% PD. The five different stress (CK, 20% PD, 30% PD, 40% PD, and 50% PD) are represented by dark blue, orange, yellow, green, and light blue, respectively. Means with different lowercase letters in the same stress time indicate there are significant differences between different treatment groups (*p* < 0.05).

Within 7 d of PEG stress, Pn, Tr, and Gs at 20–50% PD stress showed a decreasing trend compared with CK (Figure 7A–C). With more decreases at higher concentrations. Pn, Tr, and Gs increased in 20% PD and 30% PD. *Gs* increased by 8.3% and 16.7% in 20% PD and 30% PD, respectively. Fv/Fm, ΦPSII, and qP slowly decreased (Figure 7D–F). Qn slowly increased. After 4 d of PD (Figure 7G), the decreases in *Gs*, Fv/Fm, and ΦPSII increased significantly. *Ci* increased slowly with the increase in PEG concentration (Figure 7H). Pn decreased by 36.6%, 47.5%, 61.6%, and 68.5%, and Tr decreased by 28.8%, 39.4%, 53%, and 56.1% compared with CK.

### 2.6. Determination of Cell Membrane Systems, Chlorophyll Concentration, Osmotic Adjustment Substances, and Antioxidant Enzyme Activity of H. middendorffii Under PD

**Figure 8:**
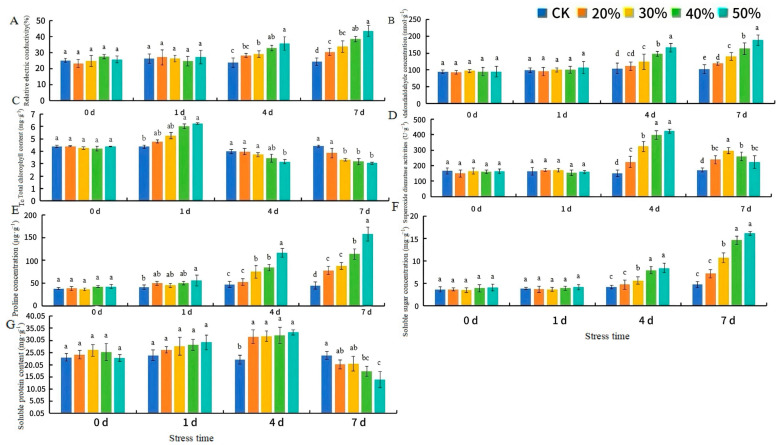
Cell membrane systems, chlorophyll concentration, osmotic adjustment substances, and antioxidant enzyme activity changes in *H. middendorffii* under PD. (**A**–**G**) Relative electric conductivity, malondialdehyde (MDA) concentration, total chlorophyll, superoxide dismutase (SOD), proline concentration (Pro), soluble sugar (SS) concentration, and soluble protein content. *H. middendorffii* leaves rapidly accumulated osmoregulatory substances within 7 d of PD. The chlorophyll content and antioxidant enzyme activities of *H. middendorffii* initially increased and then decreased within 7 d of PD. The five different stress (CK, 20% PD, 30% PD, 40% PD, and 50% PD) are represented by dark blue, orange, yellow, green, and light blue, respectively. Means with different lowercase letters in the same stress time indicate there are significant differences between different treatment groups (*p* < 0.05).

After 4 d of PD, the relative conductivity, MDA content, Pro activities, and SS content were significantly increased in the leaves of PD compared with CK (Figure 8A,B,E,F). The rate of increase was proportional to the PD concentration. After 7 d of PD, the MDA content of 20% PD and 50% PD was 16.5% and 84.0% higher than that of CK, respectively. With increasing PEG concentration, chlorophyll content was significantly decreased in the plants at 4 d of PD (Figure 8C). The 20% PD chlorophyll content was higher than that of CK. The chlorophyll content of 50% PD was the lowest, 31.1% lower than that of CK. SOD activity and soluble protein increased rapidly within 4 d of PD and decreased rapidly beyond 4 d (Figure 8D,G). The SOD activities of 20% PD and 50% PD increased by 22% and 26.2%, respectively, compared with that of CK. In addition, the Pro content and SS content of the leaves of plants in PD increased rapidly in a short period (Figure 8E,F). After 7 d of PD (Figure 8G), the soluble protein content decreased.

### 2.7. Gene Cloning and Bioinformatic Analysis of HmWRKY9

**Figure 9:**
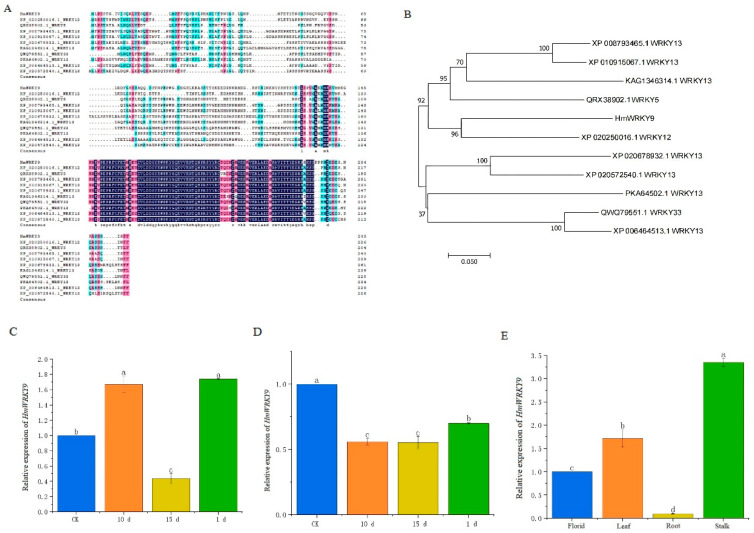
Identification and expression analysis of *HmWRKY9*. (**A**) Sequence alignment of *HmWRKY9*. (**B**) Cluster analysis of *HmWRKY9* protein with WRKY protein of other species. (**C**) The relative expression pattern of *HmWRKY9* in *H. middendorffii* leaves under ND and PD. (**D**) The relative expression pattern of *HmWRKY9* in *H. middendorffii* roots under ND and PD. (**E**) The relative expression of *HmWRKY9* in different tissues (flowers, leaves, roots, and stalk) of *H. middendorffii*.

After isolation of the *HmWRKY9* gene from *H. middendorffii*, it was characterized and bioinformatically analyzed (Figure 9A). The full-length sequences of *HmWRKY9* were 735 bp, encoding 244 amino acids. The phylogenetic tree of *HmWRKY9* from different species revealed that the amino acid sequence of *HmWRKY9* was closely related to sequences from *Lilium brownii*, *Phoenix dactylifera*, *Elaeis guineensis*, and *Cocos nucifera*. HmWRKY9 is closely related to the remaining 7 species, except for its distant affinities with 3 species, ZaWRKY33 (QWQ79551.1), AsWRKY13 (PKA64502.1), and CsWRKY13 (XP_006464513.1). The *HmWRKY9* protein sequence had the highest identity with AoWRKY12 (XP_020250016.1) (Figure 9B). The qRT-PCR analysis was performed to validate the expression of *HmWRKY9* in *H. middendorffii* roots and leaves (Figure 9C,D). Within 15 d of ND, the expression of *HmWRKY9* was consistently down-regulated in the roots. Within 10 d of ND, the expression of *HmWRKY9* in the leaves was significantly up-regulated. However, as the drought deepened, the expression of *HmWRKY9* in the leaves was significantly down-regulated at 15 d of ND. Within 1 d of PD, the expression of *HmWRKY9* was down-regulated in the roots and up-regulated in the leaves. Thus, *HmWRKY9* gene expression was up-regulated in the leaves after ND and PD.

## 3. Discussion

In actual growing environments, the effects of soil water deficit on plant growth are complex. Drought conditions can severely affect plant growth and development. Natural water loss stress and PEG-simulated stress are common methods of drought stress [30]. In this study, we examined the morphological and physiological characteristics of *H. middendorffii* under ND and PD conditions and explored the drought tolerance threshold of *H. middendorffii*.

When plants are subjected to drought stress, they first respond through changes in external morphology [31]. An elevated root–crown ratio improves the ability of plants to access water and nutrients, reflecting their drought tolerance [32]. After drought stress, the leaf length and width of *H. middendorffii* become shorter and the root–crown ratio increased significantly. In which the RWC declined rapidly after 15 d of ND. This may be due to the decline in RWC after ND 15 d, which inhibited the growth of *H. middendorffii* and maintained high levels of RWC within ND 15 d, indicating that it has strong drought resistance. In rice, a high root–crown ratio contributed to increased plant tolerance to drought stress [33]. The RWC of leaves could reflect the water condition in plants [34]. ND limited olive growth, and drought-tolerant olive cultivars were able to maintain high levels of RWC in the leaves under drought stress [35]. After exceeding the drought tolerance limit of olives, the RWC of the leaves decreased rapidly. Similar results were observed in our study. This indicated that plants could adapt to drought by increasing the root–crown ratio and leaf water content.

Photosynthesis is one of the most critical processes of growth and development for plants [36]. When subjected to drought stress, plants will maintain water supply by reducing leaf transpiration and closing stomata. Stomatal limiting factors and non-stomatal limiting factors cause decreases in plant photosynthesis. Gs is the most crucial photosynthesis parameter under abiotic stress [37]. The Pn, Tr, Gs, and Ci of *H. middendorffii* increased after ND and PD. This suggests that the loss of soil moisture did not damage *H. middendorffii* and that photosynthesis still occurred. It can be observed that when *H. middendorffii* was exposed to relatively brief periods (15 d and 4 d) of ND and PD, its stomata were enclosed to prevent water loss. With stress for 15 d, the trends for Pn, gs, and Ci exhibited a consistent decline, indicating that stomatal limitations (SLs) were the main factor in limiting photosynthesis. When PD was maintained for 4 d–7 d, the trends of Ci were in the opposite direction to those of Pn and gs. This indicated that the chloroplast structure had been further damaged and that non-stomatal limitations (NSLs) were the main factor inhibiting photosynthesis. And, the factors responsible for the decrease in photosynthesis in *H. middendorffii* at this time shifted from stomatal limiting factors to non-stomatal limiting factors. Drought stress may have caused both physiological and structural damage. This result is similar to those of Lobato et al. and Yan et al. [38,39]. Lobato et al. compared the physiology of two different pigeon pea genotypes under water deficit. The stomatal characteristics of the water deficit-tolerant genotype (I43) were less negatively affected. Yan et al. investigated the photosynthetic characteristics of blue honeysuckle under drought stress and found that Pn, Gs, Ci, and Tr decreased. The difference between the results of the present study and those of Yan et al. may be because the test materials have different drought tolerance ranges. The results of this study indicate that drought stress causes plants to close their stomata, directly inhibiting photosynthesis and reducing photosynthesis. And, the drought-tolerant varieties were less negatively affected. The maintenance of gas exchange ensured the influx of carbon dioxide in the leaf and mitigated the effects of water deficit on drought-tolerant varieties.

Chlorophyll fluorescence kinetic parameters can quickly and accurately reflect the absorption, transfer, and conversion of light energy in plants under drought stress [40]. Fv/Fm is a robust indicator of plant health, and most plant species exhibit a mean Fv/Fm of 0.83 in healthy photosynthetic tissues [41]. In this study, the Fv/Fm values of *H. middendorffii* showed a downward trend in ND and PD, indicating that the PSII of *H. middendorffii* was damaged to varying degrees under drought stress. Drought stress has caused unavoidable damage to the internal structure of the *H. middendorffii.* The reduction in light energy for carbon assimilation is accompanied by increased energy dissipation as heat due to the damage to PSII. This was well confirmed by the results of qN in this study, which increased after ND and PD. However, when the ion concentration was elevated to an excessive degree (40% PD and 50% PD) or was prolonged (15–25 d and 4–7 d), this contributed to the damage in the PSII and degradation of chlorophyll, resulting in a non-stomatal limitation for photosynthesis. The study of Yan et al. reveals that the physiological responses of *Robinia pseudoacacia*, *Amorpha fruticose*, *Medicago sativa*, and *Zea mays* species to drought are similar [42]. The photosynthesis rate is suitable for most plant species. When the Fv/Fm and NPQ began significantly declining in the woody plants, the normalized photosynthesis rate, stomatal conductance, and transpiration rate showed significant decreases [42]. Our study obtained similar results to the opinions of Yan et al. [42]. This showed that low levels of drought stress may not affect photosynthesis in plants and fully utilized the light energy by reducing qN through Fv/Fm and ΦPSII under low concentrations of PEG stress.

Chlorophyll content is essential in photosynthesis, and water deficit is an important factor in chlorophyll degradation. The changes in chlorophyll content under drought conditions reflected the sensitivity of plants to drought stress [43]. In the process of this study, the chlorophyll content of plants in ND and PD reached their maximums at 10 d and 4 d, respectively. However, in the study of the plantain tree, the chlorophyll content decreased continuously under water stress. A previous study illustrated that the chlorophyll content decreased continuously under water stress [44]. This may be because our experiments were set up with a more detailed drought stress gradient, which could capture the changes in that gradient more precisely. *H. middendorffii* has some drought resistance, leading to different results. As the drought deepened beyond the tolerance of *H. middendorffii*, the cellular structure was destroyed. The amount of synthesized chlorophyll decreased. Li et al. showed that 20% and 30% PEG drought stress caused damage at the cellular level and chloroplast lysis [45], which was similar to the results of Li et al. [45]. The results of this experiment may indicate that mild drought activates the plant protection system. It increases the content of chlorophyll a and chlorophyll b, thus maintaining normal photosynthesis. However, as drought stress intensified, the chloroplasts of plants were damaged, resulting in a gradual decrease in chlorophyll content.

When plants are subjected to drought stress, excessive ROS are produced, which need to be scavenged to maintain homeostasis in plants [46]. Osmotic regulation stabilizes the antioxidant system and reduces ROS production. MDA, SS, and Pro are critical molecules. These osmotic adjustment substances accumulate under drought stress, helping maintain a low osmotic potential inside the cells [47,48]. In this experiment, the MDA content, SOD activities, POD activities, CAT activities, SS content, and Pro content of *H. middendorffii* were increased at the beginning of ND stress and PD stress. And, the 20% PD growth increase is slight. This suggests that prolonged treatment with high concentrations of PEG caused severe damage to the cell membrane. Drought stress increased Pro content, which had been proposed to protect antioxidant enzymes and plasma membranes. Within 15 d of ND and 4 d of PD stress, *H. middendorffii* reduced ROS accumulation by increasing the activity of antioxidant enzymes. The SOD activities of *H. middendorffii* peaked at 10 d of ND and 4 d of PD, while the POD and CAT activities peaked at 15 d of ND. When the drought stress exceeds the capacity of the defense system to regulate it, the mechanism for scavenging ROS is inhibited to a certain extent. The findings of Li et al. illustrate that the Pro content of ND and PD stress continue to increase with deepening drought. PEG treatment promoted SOD, POD, and CAT activities in Cyclocarya paliurus seedlings [49]. However, this enhancement was not sufficient to prevent peroxidative damage to membrane lipids at high concentrations of PEG. When the concentration of PEG exceeds 15%, *C. paliurus* seedlings would not survive well. Vendruscolo et al. showed that producing Pro content under drought conditions reduces membrane lipid peroxidation and improves drought resistance in wheat [50]. Similar results were observed in our study. The results of this experiment may indicate that excessive drought stress disrupts intracellular ion homeostasis, inhibits antioxidant enzyme activities in plants, and reduces SS and Pro accumulation. SOD, POD, and CAT activities and MDA content were able to alleviate the damage to the cell membrane system under mild stress conditions. However, damage to the plant is significantly exacerbated when the plant’s defense system limits are exceeded. Therefore, plants can be protected from damage caused by an adverse environment by increasing osmoregulatory substances and enhancing antioxidant enzyme activities.

The WRKY TFs family is a plant-specific gene family that plays an important role in abiotic stress response [51]. SlWRKY8 is a positive regulator in *Solanum lycopersicum* resistance to pathogen infection and to drought and salt stress [52]. The maize WRKY transcription factor ZmWRKY79 positively regulates drought tolerance by increasing ABA biosynthesis [53]. There were 5 *AdWRKYs* in *Arachis duranensis* that were up-regulated after drought stress [54]. In this study, the *HmWRKY9* gene was screened and cloned based on the H. middendorffii transcriptome. HmWRKY9 is most closely related to the AoWRKY12 protein. The expression of *HmWRKY9* was up-regulated in the leaves within 15 d of ND, which suggests that *HmWRKY9* was involved in drought response. With the deepening of drought, the expression of *HmWRKY9* was significantly down-regulated in the leaves, at which point the drought level had exceeded the drought tolerance threshold of *H. middendorffii*. We also noticed a consistent down-regulation of *HmWRKY9* expression in the roots under ND, which may be related to the tissue-specific expression of *HmWRKY9*. The expression of the *HmWRKY9* was mainly in the leaves and was lowest in the roots.

## 4. Materials and Methods

### 4.1. Plant Materials

The original *H. middendorffii* seedlings were obtained by division from the ornamental plant resource nursery at Jilin Agricultural University, Changchun, China (43.49′1.283″ N, 125.240′33.964″ E). One-year-old seedlings with robust growth and uniform growth status were selected and transplanted into plastic pots of the same size and texture. The substrate for cultivating seedlings was prepared by mixing them with peat and perlite in a ratio of 3:1, and the seedlings were then cultivated for 4 weeks. A total of 144 pots of seedlings were raised.

### 4.2. Natural Drought Stress and PEG-Simulated Drought Stress of Seedlings

Natural drought stress is divided into CK and natural drought stress (ND). The CK group was irrigated with 200 mL of distilled water every 3 d, and the ND group was treated for 25 d using the natural water loss method. The leaves were taken in the same position during the treatment for 0, 5, 10, 15, 20, and 25 d. The PEG-simulated drought stress is divided into CK and PEG drought stress (20% PD, 30% PD, 40% PD, and 50% PD). PD was irrigated with 200 mL of (20%, 30%, 40%, and 50%) PEG-6000 every 3 d. The leaves were taken in the same position during the treatment for 0, 1, 4, and 7 d. After flash freezing with liquid nitrogen (Ruide, Changchun, China), the samples were stored in a −80 °C freezer (TSE320V-ULTS, Thermofisher, Waltham, MA, USA) for analysis.

### 4.3. Determination of Morphological and Physiological Indicators

The height of the plant, the width of the leaves, and the width of the crown were measured. The plants were divided into above-ground and below-ground parts based on the contact surface of the main stem of seedlings with the soil [55]. The root–shoot ratio (R:S) was then calculated. The leaf relative water content was determined using the drying constant weight method. The acetone-ethanol method was employed to extract the chlorophyll concentration [56]. The conductivity method was used to measure the sample’s relative conductivity using an electronic conductivity meter (FE30, Mettler-Toledo International Inc, Shanghai, China). MDA concentration was measured using an MDA extraction kit (BC0020, Beijing Solarbio Science & Technology Co., Ltd., Beijing, China). SOD activity was measured with an SOD activity assay kit (BC5165, Beijing Solarbio Science & Technology Co., Ltd., Beijing, China). The concentration of Pro was measured with a Pro extraction assay kit (P9460, Beijing Solarbio Science & Technology Co., Ltd., Beijing, China). CAT activity was measured with a CAT activity assay kit (C8070, Beijing Solarbio Science & Technology Co., Ltd., Beijing, China). The concentration of Pro was measured with a Pro extraction assay kit (P9460, Beijing Solarbio Science & Technology Co., Ltd., Beijing, China). SS concentration was measured according to the SS extraction assay kit (BC0030, Beijing Solarbio Science & Technology Co., Ltd., Beijing, China). The determination of soluble protein was performed following the Komas Brilliant Blue G-250 staining method [57].

### 4.4. Determination of Photosynthetic Parameters and Chlorophyll Fluorescence Parameters

A portable photosynthetic apparatus (CIRAS-2, PP system, Amesbury, MA, USA) was used for measuring the photosynthetic parameters from 8:30 am to 11:30 am on a distinctly cloudless and windless day. Then, the third functional leaf from the top was identified. The photosynthetic parameters measured included Pn, Tr, Gs, Ci, and water use efficiency (WUE). The light intensity of the instrument was set to 1200 µmol·m^−2^·s^−1^ [57], the gas flow rate of the sample chamber was set to 500 µmol·s^−1^, and the carbon dioxide concentration was set to 400 µmol·m^−1^. Each treatment was replicated 3 times, and 5 blades were determined.

The selected whole blades were dark-acclimatized for 40 min using aluminum foil-wrapped shelters. The chlorophyll fluorescence parameters were measured using the chlorophyll fluorescence imaging system (Li-6800, LI-COR, Lincoln, NE, USA). The fluorescence origin (Fo), fluorescence maximum (Fm), and maximum photochemical efficiency of PSII (Fv/Fm) were observed. The photochemical quenching coefficient (qP), non-photochemical quenching coefficient (NPQ), and quantum yield of PSII (ΦPSII) were measured. The carbon dioxide concentration was set to 450 µmol·m^−1^, and the photosynthetically active radiation of the instrument was set to 1000 µmol·m^−2^·s^−1^. Each treatment was replicated 3 times.

### 4.5. Gene Cloning and Bioinformatic Analysis of HmWRKY9

Total RNA was extracted from the leaves of *H. middendorffii*, and a PrimeScript RT reagent kit was employed to synthesize the first strand of cDNA. The product obtained by RT-PCR with a pair of primers (*HmWRKY9* upstream primer 5′-AAGAGATGTTGCCTTCCAGTACT-3′ and downstream primer 5′-CGACAACTGCAATGATCATACTA) was ligated to the pCloneEZ-TOPO vector, transformed into Escherichia coli, and then sequenced.

An online analysis of the signal peptides, phosphorylation sites, transmembrane domains, hydrophilicity analysis, secondary structure of the proteins, and the weight of the *HmWRKY9* protein amino acids in *H. middendorffii* was performed using the SignalP5.0, NetPhos3.1, TMHMM, ProtScale, Npsa, and WoLFPSORT tools. In order to study the evolutionary relationship of the *HmWRKY9* genes, MEME (https://meme-suite.org/meme/tools/meme) was used to analyze the conserved domains of the WRKY genes online, accessed on 8 October 2022, ClustalW was used to perform multiple sequence alignments on the *H. middendorffii* and *Arabidopsis* WRKY gene families, the results were merged into MEGA7.0 software, and a phylogenetic tree of the *Arabidopsis* and *H. middendorffii* WRKY gene families was constructed via the neighbor-joining method. Verification of the parameter bootstrapping was repeated 1000 times.

### 4.6. Real-Time Quantitative PCR Analyses

Leaf total RNA was extracted using the TransZol Up Plus RNA Kit (ER501-01-V2, TransGen Biotech, Beijing, China) and reverse transcribed to cDNA using the NovoScript^®^Plus All-in-one 1st Strand cDNA Synthesis (E047-01B, Novoprotein, Shanghai, China) instruction manual as a template for real-time quantitative PCR (qPCR). The qPCR was performed according to the NovoStart^®^SYBR qPCR SuperMix Plus instruction manual, and 3 biological replicates were performed for each condition.

The reaction procedure was performed pre-denaturation as follows: 1 min 95 °C, followed by 40 cycles of 95 °C for 20 s, 60 °C for 20 s, and 72 °C for 30 s. Each 20 μL of reaction mix included 10 μL of 2 × SYBR Green Real-Time PCR master Mix, 2 μL of cDNA, 1 μL of Primer−F, 1 μL of Primer−R, and 6 μL of ddH_2_O. The HfEF-1α gene (GenBank accession number: MT096368) was used as the internal gene, and the expression level in leaves without any treatment was used as a control (0 h). The relative expression of the genes was analyzed using the 2^−ΔΔCt^ method. Three biological replicates were performed for each sample. The specific primers used for the *HmWRKY9* gene were upstream primer 5′-GGAGGATGCTCGAATGGTGAT-3′ and downstream primer 5′-TGTGAGATGAAGCCCTGTTGC-3′. Those for the HfEF-1α gene was upstream primer 5′-TCTCGCCGCCTCTCTCAAT-3′ and downstream primer 5′-TTCAGCAGCTTCCTTCTCG-3′.

### 4.7. Construction of Expression Vectors and Generation of Transgenic Tobacco

The plasmid *HmWRKY9* was amplified with primers to add restriction sites for the restriction enzymes. The constructed vector pBI121-HmWRKY9-GUS was transformed into *Agrobacterium* GV3101. Tobacco transformation was performed using *Agrobacterium*-mediated transformation of tobacco leaf discs. The positive transformant lines were identified by PCR.

### 4.8. Statistical Analysis

Three replicates were set up for each treatment, and their means and standard deviations were calculated. SPSS 20.0 for Windows (SPSS Inc., Chicago, IL, USA) was used for independent sample *t*-tests. Data visualization was conducted with Origin2022.

## 5. Conclusions

In this study, we evaluated *H. middendorffii* tolerance thresholds to ND and PD. *H. middendorffii* was able to maintain a normal growth condition within 15 d of ND and 4 d of 20% PD. This provides a practical approach to water management of *H. middendorffii* in cultivation and application. *H*. *middendorffii* protected itself from damage and enhanced drought tolerance mainly by increasing the content of osmoregulatory substances, enhancing the activity of antioxidant enzymes and inhibiting photosynthesis. In addition, the expression of *HmWRKY9* under drought stress was verified, and the *HmWRKY9* gene was involved in drought regulation. This provides a theoretical basis for the subsequent cultivation of *H. middendorffii* for drought resistance, which is of great significance for the cultivation of drought-tolerant ornamental plant resources.

## Figures and Tables

**Table 1:** Classification of morphological drought resistance index of *H. middendorffii* under ND.

Drought Resistance Classification	Morphological Changes in Drought Stress
I	The entire plant thrives with a green color.
II	The outer layer begins to wilt and wither, and the tip and edge of leaves are coked.
III	The middle leaves wither and droop, the outer leaves are scorched and dry, and the plant loses its ornamental value.

**Table 2:** Effects of growth of *H. middendorffii* under ND.

Stress Time (d)	Leaf Length (cm)		Leaf Width (cm)		Crown Width (cm)	
	CK	ND	CK	ND	CK	ND
0	25.10 ± 3.22 a	27.17 ± 4.05 a	0.65 ± 0.47 a	0.61 ± 0.10 a	25.87 ± 0.95 a	26.13 ± 1.08 a
5	27.90 ± 1.13 a	29.57 ± 0.61 a	0.86 ± 0.15 a	0.87 ± 0.90 a	26.20 ± 5.73 a	26.63 ± 3.33 a
10	30.23 ± 2.61 a	30.97 ± 2.29 a	0.91 ± 0.10 a	1.05 ± 0.18 a	26.57 ± 3.86 a	29.23 ± 5.14 a
15	32.10 ± 2.00 a	32.20 ± 0.66 a	0.96 ± 0.11 b	1.14 ± 0.10 a	27.30 ± 3.34 a	34.17 ± 10.53 a
20	33.67 ± 0.61 a	34.27 ± 3.02 a	0.96 ± 0.38 a	0.91 ± 0.07 a	29.33 ± 3.55 b	36.77 ± 11.64 a
25	38.13 ± 1.91 a	35.50 ± 4.55 b	0.98 ± 0.01 a	0.87 ± 0.06 b	32.33 ± 2.83 b	45.27 ± 2.28 a

Note: Means with different lowercase letters in the same stress time indicate there are significant differences between different treatment groups (*p* < 0.05).

**Table 3:** Effects of biomass of *H. middendorffii* under ND.

Stress Time (d)	Above-Ground Fresh Weight (g)		Below-Ground Fresh Weight (g)		Above-Ground Dry Weight (g)		Below-Ground Dry Weight (g)	
	CK	ND	CK	ND	CK	ND	CK	ND
0	6.42 ± 0.28 a	6.83 ± 0.86 a	44.92 ± 0.10 a	45.63 ±5.02 a	1.61 ± 0.03 a	1.67 ± 0.05 a	10.82 ± 0.54 a	11.36 ± 0.95 a
5	6.55 ± 0.15 a	6.61 ± 0.77 a	38.44 ± 1.58 a	40.21 ±2.04 a	1.38 ± 0.2 a	1.03 ± 0.13 a	8.20 ± 0.57 a	7.95 ± 0.26 a
10	7.91 ± 0.15 a	3.75 ± 0.7 b	47.73 ± 8.78 a	28.20 ±2.63 b	1.54 ± 0.07 a	0.66 ± 0.21 b	11.24 ± 0.38 a	5.49 ± 1.18 b
15	9.63 ± 1.31 a	2.68 ± 0.61 b	43.38 ± 6.58 a	25.19 ±5.19 b	1.87 ± 0.09 a	0.47 ± 0.1 b	12.58 ± 0.25 a	4.68 ± 0.89 b
20	8.46 ± 0.20 a	2.32 ± 0.78 b	46.45 ± 0.86 a	24.04 ±1.48 b	1.54 ± 0.13 a	0.31 ± 0.03 b	11.34 ± 0.19 a	4.54 ± 0.47 b
25	9.52 ± 0.26 a	1.65 ± 0.06 b	46.76 ± 2.68 a	20.15 ±1.80 b	1.78 ± 0.17 a	0.32 ± 0.08 b	11.98 ± 0.33 a	4.94 ± 0.31 b

Note: Means with different lowercase letters in the same stress time indicate there are significant differences between different treatment groups (*p* < 0.05).

**Table 4:** Effects of morphological indicators of *H. middendorffii* under PD.

Stress Time (d)	PEG-6000 (%)	Height (cm)	Leaf Width (cm)	Crown Width (cm)
0	CK	31.43 ± 6.99 a	1.10 ± 0.21 a	36.17 ± 6.93 a
	20%	33.47 ± 2.65 a	1.03 ± 0.13 a	37.20 ± 2.00 a
	30%	31.70 ± 2.04 a	1.06 ± 0.20 a	34.01 ± 7.07 a
	40%	35.37 ± 9.33 a	1.04 ± 0.18 a	32.70 ± 3.18 a
	50%	31.37 ± 2.83 a	1.06 ± 0.23 a	35.23 ± 2.90 a
1	CK	32.93 ± 5.78 a	1.13 ± 0.22 a	38.40 ± 0.95 a
	20%	36.43 ± 2.02 a	1.10 ± 0.27 a	39.13 ± 2.85 a
	30%	34.50 ± 8.24 a	1.16 ± 0.90 a	36.20 ± 6.93 a
	40%	36.80 ± 1.04 a	1.05 ± 0.17 a	38.63 ± 2.01 a
	50%	32.30 ± 0.85 a	1.03 ± 0.20 a	39.67 ± 0.81 a
4	CK	37.57 ± 4.90 a	1.11 ± 0.22 a	39.60 ± 0.36 b
	20%	36.87 ± 0.59 ab	1.09 ± 0.27 a	40.60 ± 4.66 b
	30%	35.43 ± 7.27 ab	0.87 ± 0.54 ab	39.40 ± 0.26 b
	40%	37.90 ± 0.72 a	0.88 ± 0.62 ab	40.30 ± 1.81 b
	50%	32.53 ± 1.76 b	0.81 ± 0.38 b	43.93 ± 6.34 a
7	CK	39.80 ± 0.79 a	1.21 ± 0.94 a	40.67 ± 1.70 b
	20%	38.20 ± 0.82 a	1.02 ± 0.21 ab	41.37 ± 4.07 b
	30%	35.68 ± 1.80 b	0.80 ± 0.77 c	40.77 ± 3.69 b
	40%	38.03 ± 1.97 a	0.83 ± 0.34 bc	44.87 ± 4.04 a
	50%	32.70 ± 1.11 c	0.76 ± 0.27 c	47.01 ± 1.19 a

Note: Means with different lowercase letters in the same stress time indicate there are significant differences between different treatment groups (*p* < 0.05).

## Data Availability

The original contributions presented in the study are included in this article, and further inquiries can be directed to the corresponding authors.

## References

[1] Ghaffar A., Hussain N., Ajaj R., Shahin S.M., Bano H., Javed M., Khalid A., Yasmin M., Shah K.H., Zaheer M. (2023). Photosynthetic activity and metabolic profiling of bread wheat cultivars contrasting in drought tolerance. Front. Plant Sci..

[2] Ahmad D.M., Kam J. (2024). Disparity between global drought hazard and awareness. npj Clean Water.

[3] Gliesch M., Sanchez L.H., Jongepier E., Martin C., Hu Y., Tietema A., de Vries F.T. (2024). Heathland management affects soil response to drought. J. Appl. Ecol..

[4] Kunert K.J., Vorster B.J., Fenta B.A., Kibido T., Dionisio G., Foyer C.H. (2016). Drought stress responses in soybean roots and nodules. Front. Plant. Sci..

[5] Kumar S., Sachdeva S., Bhat K., Vats S. (2018). Plant responses to drought stress: Physiological, biochemical and molecular basis. Biotic and Abiotic Stress Tolerance in Plants.

[6] Ranjan A., Sinha R., Singla-Pareek S.L., Pareek A., Singh A.K. (2022). Shaping the root system architecture in plants for adaptation to drought stress. Physiol. Plant.

[7] Sun X., Shi J., Ding G. (2017). Combined effects of arbuscular mycorrhiza and drought stress on plant growth and mortality of forage sorghum. Appl. Soil. Ecol..

[8] Benjamin J., Nielsen D. (2006). Water deficit effects on root distribution of soybean, field pea and chickpea. Field Crops. Res..

[9] Jafari S., Garmdareh S.E.H., Azadegan B. (2019). Effects of drought stress on morphological, physiological, and biochemical characteristics of stock plant (*Matthiola incana* L.). Sci. Hortic..

[10] Yang Z., Zhou B., Chen Q., Ge X., Wang X., Cao Y., Tong R., Shi Y. (2018). Effects of drought on root architecture and non-structural carbohydrate of *Cunninghamia lanceolata*. Acta Ecol. Sin..

[11] Mathobo R., Marais D., Steyn J.M. (2017). The effect of drought stress on yield, leaf gaseous exchange and chlorophyll fluorescence of dry beans (*Phaseolus vulgaris L.*). Agr. Water Manag..

[12] Abreha K.B., Enyew M., Carlsson A.S., Vetukuri R.R., Feyissa T., Motlhaodi T., Ng’uni D., Geleta M. (2022). Sorghum in dryland: Morphological, physiological, and molecular responses of sorghum under drought stress. Planta.

[13] Silva D.M.R., Barros A.C., Silva R.B., Galdino W.d.O., Souza J.W.G.d., Marques I.C.d.S., Sousa J.I.d., Lira V.d.S., Melo A.F., Abreu L.d.S.d. (2024). Impact of Photosynthetic Efficiency on Watermelon Cultivation in the Face of Drought. Agronomy.

[14] Gao H., Bai N., Zhang Y., Zhang X., Zhang Y., Wang L., Wang E., Tian Y., Guo Y., Yan F. (2022). Drought stress alters gas exchange, chlorophyll fluorescence, and antioxidant enzyme activities in Glycyrrhiza uralensis in the Hexi Corridor, China. Russ. J. Plant Physiol..

[15] Ping M., Bai T.-H. (2015). Effects of progressive drought on photosynthesis and partitioning of absorbed light in apple trees. J. Integre. Agric..

[16] Marshall J., Rutledge R., Blumwald E., Dumbroff E. (2000). Reduction in turgid water volume in jack pine, white spruce and black spruce in response to drought and paclobutrazol. Tree Physiol..

[17] Seleiman M.F., Al-Suhaibani N., Ali N., Akmal M., Alotaibi M., Refay Y., Dindaroglu T., Abdul-Wajid H.H., Battaglia M.L. (2021). Drought stress impacts on plants and different approaches to alleviate its adverse effects. Plants.

[18] Basu S., Ramegowda V., Kumar A., Pereira A. (2016). Plant adaptation to drought stress. F1000Research.

[19] Miller G., Suzuki N., Ciftci-Yilmaz S., Mittler R. (2010). Reactive oxygen species homeostasis and signalling during drought and salinity stresses. Plant Cell Environ..

[20] Apel K., Hirt H. (2004). Reactive oxygen species: Metabolism, oxidative stress, and signal transduction. Annu. Rev. Plant Biol..

[21] Liu L., Cao X., Zhai Z., Ma S., Tian Y., Cheng J. (2022). Direct evidence of drought stress memory in mulberry from a physiological perspective: Antioxidative, osmotic and phytohormonal regulations. Plant Physiol. Biochem..

[22] Sutulienė R., Brazaitytė A., Małek S., Jasik M., Samuolienė G. (2023). Response of oxidative stress and antioxidant system in pea plants exposed to drought and boron nanoparticles. Antioxidants.

[23] Cao Y., Yang W., Ma J., Cheng Z., Zhang X., Liu X., Wu X., Zhang J. (2024). An Integrated Framework for Drought Stress in Plants. Int. J. Mol. Sci..

[24] Liu H., Bao G., Dou Z., Liu H., Bai J., Chen Y., Yuan Y., Zhang X., Xi J. (2022). Response characteristics of highland barley under freeze-thaw, drought and artemisinin stresses. BMC Plant Biol..

[25] Babitha K., Ramu S., Pruthvi V., Mahesh P., Nataraja K.N., Udayakumar M. (2013). Co-expression of at *bHLH17* and at *WRKY 28* confers resistance to abiotic stress in Arabidopsis. Transgenic Res..

[26] Wang X., Du B., Liu M., Sun N., Qi X. (2013). Arabidopsis transcription factor *WRKY33* is involved in drought by directly regulating the expression of *CesA8*. Am. J. Plant Sci..

[27] Che Y., Sun Y., Lu S., Zhao F., Hou L., Liu X. (2018). *AtWRKY40* functions in drought stress response in Arabidopsis thaliana. Plant Physiol. J..

[28] Lee H., Cha J., Choi C., Choi N., Ji H.-S., Park S.R., Lee S., Hwang D.-J. (2018). Rice *WRKY11* plays a role in pathogen defense and drought tolerance. Rice.

[29] Wang C.-T., Ru J.-N., Liu Y.-W., Yang J.-F., Li M., Xu Z.-S., Fu J.-D. (2018). The maize WRKY transcription factor *ZmWRKY40* confers drought resistance in transgenic *Arabidopsis*. Int. J. Mol. Sci..

[30] Ahmad M.A., Javed R., Adeel M., Rizwan M., Yang Y. (2020). PEG 6000-stimulated drought stress improves the attributes of in vitro growth, steviol glycosides production, and antioxidant activities in *Stevia rebaudiana* Bertoni. Plants.

[31] Sintaha M., Man C.K., Yung W.S., Duan S., Li M.W., Lam H.M. (2022). Drought stress priming improved the drought tolerance of soybean. Plants.

[32] Rowland L., Oliveira R.S., Bittencourt P.R., Giles A.L., Coughlin I., Costa P.d.B., Domingues T., Ferreira L.V., Vasconcelos S.S., Junior J.A. (2021). Plant traits controlling growth change in response to a drier climate. New Phytol..

[33] Guo Y., Du Y., Niu X., Ma Y., Song G., Cao C., Li P., Chen Y., Siddique K.H. (2024). Comparison of Drought Stress Responses in Large-and Small-Rooted Rice Lines: Physiological, Anatomical, and Hormonal Changes. J. Plant Growth Regul..

[34] Chen X., Zhu Y., Ding Y., Pan R., Shen W., Yu X., Xiong F. (2021). The relationship between characteristics of root morphology and grain filling in wheat under drought stress. PeerJ.

[35] Karimi S., Rahemi M., Rostami A.A., Sedaghat S. (2018). Drought effects on growth, water content and osmoprotectants in four olive cultivars with different drought tolerance. Int. J. Fruit Sci..

[36] Zhao N., Meng P., Yu X. (2019). Photosynthetic stimulation of saplings by the interaction of CO_2_ and water stress. J. For. Res..

[37] Bo W., Fu B., Qin G., Xing G., Wang Y. (2017). Evaluation of drought resistance in *Iris germanica* L. based on subordination function and principal component analysis. Emir. J. Food Agric..

[38] Lobato S.M.d.S., dos Santos L.R., da Silva B.R.S., Melo W.d.O., Lobato A.K.d.S. (2021). Protective mechanism triggered by Pigeonpea plants exposed to water deficit: Modifications linked to paraheliotropism, stomatal characteristics and antioxidant enzymes. J. Plant Growth Regul..

[39] Yan W., Lu Y., Guo L., Liu Y., Li M., Zhang B., Zhang B., Zhang L., Qin D., Huo J. (2024). Effects of Drought Stress on Photosynthesis and Chlorophyll Fluorescence in Blue Honeysuckle. Plants.

[40] Jin E.J., Yoon J.-H., Lee H., Bae E.J., Yong S.H., Choi M.S. (2023). Evaluation of drought stress level in Sargent’s cherry (*Prunus sargentii* Rehder) using photosynthesis and chlorophyll fluorescence parameters and proline content analysis. PeerJ.

[41] Wang J., Zhang X., Han Z., Feng H., Wang Y., Kang J., Han X., Wang L., Wang C., Li H. (2022). Analysis of physiological indicators associated with drought tolerance in wheat under drought and re-watering conditions. Antioxidants.

[42] Yan W.M., Zhong Y.Q., Shangguan Z.P. (2017). Responses of different physiological parameter thresholds to soil water availability in four plant species during prolonged drought. Agric. For. Meteorol..

[43] Chen J., Liu X., Du S., Ma Y., Liu L. (2021). Effects of drought on the relationship between photosynthesis and chlorophyll fluorescence for maize. IEEE J. Sel. Top Appl. Earth Obse. Remote Sens..

[44] Akhbarfar G., Nikbakht A., Etemadi N., Gailing O. (2023). Physiological and Biochemical Responses of Plantain Trees (*Platanus orientalis* L.) Derived from Different Ages to Drought Stress and Ascophyllum nodosum L. Extract. J. Soil Sci. Plant Nutr..

[45] Li B., Feng Y., Zong Y., Zhang D., Hao X., Li P. (2020). Elevated CO_2_-induced changes in photosynthesis, antioxidant enzymes and signal transduction enzyme of soybean under drought stress. Plant Physiol. Biochem..

[46] Dong S., Zhou Q., Yan C., Song S., Wang X., Wu Z., Wang X., Ma C. (2023). Comparative protein profiling of two soybean genotypes with different stress tolerance reveals major components in drought tolerance. Front. Sus. Food Syst..

[47] Gomes F.P., Oliva M.A., Mielke M.S., Almeida A.-A.F., Aquino L.A. (2010). Osmotic adjustment, proline accumulation and cell membrane stability in leaves of Cocos nucifera submitted to drought stress. Sci. Hortic..

[48] Li C., Wan Y., Shang X., Fang S. (2022). Responses of microstructure, ultrastructure and antioxidant enzyme activity to PEG-induced drought stress in *Cyclocarya paliurus* seedlings. Forests.

[49] Vendruscolo E.C.G., Schuster I., Pileggi M., Scapim C.A., Molinari H.B.C., Marur C.J., Vieira L.G.E. (2007). Stress-induced synthesis of proline confers tolerance to water deficit in transgenic wheat. J. Plant Physiol..

[50] Rushton P.J., Somssich I.E., Ringler P., Shen Q.J. (2010). WRKY transcription factors. Trends Plant Sci..

[51] Gao Y.F., Liu J.K., Yang F.M., Zhang G.Y., Wang D., Zhang L., Ou Y.B., Yao Y.A. (2020). The WRKY transcription factor WRKY8 promotes resistance to pathogen infection and mediates drought and salt stress tolerance in *Solanum lycopersicum*. Physiol. Plant..

[52] Gulzar F., Fu J., Zhu C., Yan J., Li X., Meraj T.A., Shen Q., Hassan B., Wang Q. (2021). Maize WRKY Transcription factor *ZmWRKY79* positively regulates drought tolerance through elevating ABA biosynthesis. Int. J. Fruit Sci..

[53] Zhang Y., Du P., Xiong F., Zhang X., Song H. (2022). WRKY genes improve drought tolerance in *Arachis duranensis*. Front. Plant Sci..

[54] Yu X., Liu Y., Cao P., Zeng X., Xu B., Luo F., Yang X., Wang X., Wang X., Xiao X. (2023). Morphological structure and physiological and biochemical responses to drought stress of *Iris japonica*. Plants.

[55] Ngcobo S., Bada S.O., Ukpong A.M., Risenga I. (2024). Optimal chlorophyll extraction conditions and postharvest stability in Moringa (*M. Oleifera*) leaves. J. Food Meas. Charact..

[56] Zhang F., Yu J., Johnston C.R., Wang Y., Zhu K., Lu F., Zhang Z., Zou J. (2015). Seed priming with polyethylene glycol induces physiological changes in sorghum (*Sorghum bicolor* L. Moench) seedlings under suboptimal soil moisture environments. PLoS ONE.

[57] Santanoo S., Lontom W., Dongsansuk A., Vongcharoen K., Theerakulpisut P. (2023). Photosynthesis performance at different growth stages, growth, and yield of rice in saline fields. Plants.

